# Effect of autologous conditioned serum (ACS) on histological characteristics and expression of soft tissue IL-1β gene after horizontal ridge augmentation surgery

**DOI:** 10.34172/japid.2023.011

**Published:** 2023-06-10

**Authors:** Mehdi Mojtahedi Nia, Adileh Shirmohammadi, Hamidreza Mohammadi, Leila Roshangar, Amirreza Babaloo

**Affiliations:** ^1^Department of Periodontics, Faculty of Dentistry, Tabriz University of Medical Sciences, Tabriz, Iran; ^2^Department of Histology and Embryology, Faculty of Medicine, Tabriz University of Medical Sciences, Tabriz, Iran

**Keywords:** Alveolar ridge augmentation, Dental implant, Novel interleukin receptor

## Abstract

**Background.:**

Horizontal ridge augmentation surgeries are common procedures in periodontics. Histological changes in soft tissues affect the success of surgery in many ways. Autologous conditioned serum (ACS) increases interleukin-1 receptor antagonist (IL-1Ra) and causes inflammation modulation. Therefore, the present study aimed to investigate the effect of ACS on histological changes and gene expression of soft tissues after horizontal ridge augmentation surgeries.

**Methods.:**

This double-blind split-mouth clinical trial was performed on patients needing implants with horizontal ridge augmentation (n=21). The control and intervention groups were considered split-mouth in two areas of the patients’ oral soft tissues. A collagen membrane impregnated with ACS was used on the test side, and only a collagen membrane was used on the control side. After four months, histological changes in soft tissues, such as the amount of connective tissue collagen, fibroblast and inflammatory cell counts, and expression of the IL-1β gene, were evaluated.

**Results.:**

The fibroblast counts in the ACS group were significantly higher than in the control group. In addition, ACS caused a significant increase in the amount of collagen in the soft tissues compared to the control group (*P*<0.01). However, the number of inflammatory cells was similar in the two groups (*P*>0.05). IL-1β gene expression was not significantly different between the case and control groups.

**Conclusion.:**

Under the limitations of the present study and based on the results of histological examinations, ACS increased the number of fibroblasts and the amount of collagen in soft tissues without affecting inflammatory cells (*P*=0.562).

## Introduction

 Teeth are extracted for various reasons, including caries and advanced periodontal disease. After tooth extraction, the dimensions of the edentulous ridge decrease due to remodeling in the vertical and horizontal dimensions. As a result, it is difficult to place an implant in the area. Therefore, by using preventive surgical techniques such as socket preservation during tooth extraction, the amount of remodeling can be reduced to increase the horizontal dimension in the area. The horizontal ridge augmentation technique can be used to this end.^1,2^

 The wound healing process consists of four stages: homeostasis, inflammation, repair, and remodeling. The hemostatic phase begins with tissue damage after surgery.^3^ Blood clots rapidly occupy the site of injury. Growth factors in the clot trigger inflammatory cells, including neutrophils and macrophages in the area, which clear the wound. The formation phase of new tissues begins with the formation of “granular tissue,” a morphological term that refers to the highly vascular tissue composed of fibroblasts and extracellular matrix. Many cytokines and growth factors, such as the TGF-β and interleukin (IL) family and angiogenesis factors, participate in this stage.^4,5^ The maturation phase is also called the remodeling phase. This is when type III collagen changes to type I, and the wound closes completely. The long-term remodeling phase causes scar tissue formation. In addition to esthetic reasons, scar tissue has less biomechanical capacity. Scar tissue formation, also called fibrosis, is the main pathological outcome of various injuries associated with long-term inflammation. Therefore, considerable effort is made to control scarring.^6^ For example, subepithelial connective tissue grafts remain stable in the long term.^6,7^ Therefore, it is reasonable to say that dense and stable soft tissues can have clinical benefits. Autologous conditioned serum (ACS) is a type of serum taken from a person’s own blood sample. It seems that using the individual’s own serum is most compatible with the in vivo phenomenon and the cell that can have favorable effects.^8^ Using human blood, a 140-fold increase was reported in the interleukin-1 receptor antagonist (IL-1Ra).^9^ In ACS, macrophages and monocytes are the major endogenous sources for IL-1Ra.^10,11^ Various stimuli, including adhesion to specific surfaces, can increase IL-1Ra production.^10^ Based on this information, Meijer et al^12^ developed a method to stimulate the synthesis of IL-1Ra by whole human blood. According to their method, blood is drawn into a syringe containing glass beads coated with CrSO4, to which blood monocytes and other adherent cells have the opportunity to attach. This blood product was first used to treat arthritis, and significant success indicates that ACS modulates inflammation by providing IL-1Ra, preventing the destructive effects of over-inflammation.^13^ The present study investigated the effect of ACS on the histological characteristics of soft tissues, including collagen and fibroblasts, the number of inflammatory cells, and the expression of the IL-1β gene in soft tissues after horizontal ridge augmentation surgeries.

## Methods

 In this split-mouth study, 21 patients referring to the Periodontics Department of the Dental Clinic of Tabriz University of Medical Sciences were included.

###  Inclusion criteria

 The difficulty of surgery based on the dimensions required for augmentation and the condition of the existing tooth before extraction and the ridge condition of one side compared to the other side was the same; patients required implant placement in the area and were willing to undergo surgery.

###  Exclusion criteria

 Use of drugs interfering with the treatment process, the presence of systemic diseases, smoking, inflammation or infection or pain during surgery, a recent history of non-steroidal inflammatory drug use, patients with gastrointestinal problems and allergies, use of NSAIDs, and pregnancy.

 After psychologically preparing the volunteer patient and obtaining written consent, CBCT radiographic examinations were carried out to determine the need for surgery, and radiographic and clinical evaluations were performed before the operation. A blinded parallel-groups randomized clinical trial was performed.

###  ACS preparation 

 One day before surgery, 10 mL of blood was taken from the patient’s vein and immediately injected into a special ACS syringe. The syringe was incubated at 37 °C for 8 hours and then centrifuged at 3000 rpm for 10 minutes. After centrifugation, the syringe was retrieved from the machine; it consisted of two upper and lower parts. The upper part was carefully removed from the syringe, stored as ACS in the refrigerator, and used on the day of operation.

###  Horizontal ridge reconstruction surgery

 After local anesthetic agent injection and ensuring anesthesia, the patient underwent horizontal bone augmentation surgery. The crestal incision was created using a #15 blade, and flap elevation was performed. One milliliter of graft material (FDBA) was impregnated with 1 mL of prepared ACS and placed in the area, and an ACS-impregnated Bio-Gide collagen membrane was used to cover the area. The reflected soft tissue was returned and sutured using 3-0 silk thread. The control side underwent the same surgical protocol without using ACS. Four months after surgery, the area for implant placement was evaluated. A CBCT radiographic evaluation of the area was performed.

###  Histological evaluation

 To examine histological changes, the soft tissue of the area was punched to the diameter of the implant, immediately transferred to a container containing 10% formalin buffer, and sent to the histology laboratory. After tissue passage, routine and specific staining were performed using hematoxylin-eosin and Sirius Red F3Ba staining methods. Six areas were selected from each section, and digital images were taken under a microscope at a magnification of × 40. No difference between intervention and control groups was reported.

###  Evaluation of IL-1 gene expression

 To investigate the expression of the IL-1 gene, the samples were immersed in RNas inhibitor solution immediately after sampling to fix mRNA, transferred to a -70 °C freezer, and evaluated using an mRNA extraction kit (Invitek kit, Germany). Total biopsies were extracted. Total RNA was measured using a spectrophotometer, and then 3 µL of total RNA was converted to cDNA using the RevertAid First cDNA synthesis kit. Special primers and total mean probes were designed using Primer Express (TAG Copenhagen) software. All real-time PCR reactions were performed on Rotor-Gene TM 3000 (Corbett).

###  Statistical analysis

 Descriptive statistical methods were used to report data [(mean ± standard deviation and frequencies (percentages)]. To compare the amount of bone formed in the study groups, paired t-test or an equivalent non-parametric test was used. SPSS 21 was used for data analysis. In this study, a *P* < 0.05 was considered significant.

## Results

 In this study, 21 patients required horizontal ridge augmentation; 58% and 42% of the subjects were male and female, respectively, with 12 patients undergoing surgery in the upper jaw and 9 in the lower jaw. As shown in Table 1 according to histological assessments, collagen levels and fibroblasts were significantly different between the case and control groups (*P* ≤ 0.05). However, there was no significant difference in the number of inflammatory cells between the case and control groups (*P*≥ 0.05). In addition, as shown in Table 2, IL-1β gene expression showed no significant difference between the case and control groups (*P*≥ 0.05).

**Table 1:** Comparison of fibroblasts, inflammatory cells, and soft tissue collagen after horizontal ridge reconstruction surgery in the control and ACS groups

**Group**	**Number**	**Fibroblast counts**	**Inflammatory cell counts**	**Amount of collagen**
Control group	(n = 21)	42 ± 2.8	12.2 ± 4.4	60 ± 1.8
ACS group	(n = 21)	56 ± 4.2	11.8 ± 5	68 ± 3.1
*P* value	-	< 0.01	> 0.05	< 0.01

**Table 2:** Comparison of IL-1β gene expression between the case and control groups

**Group**	**Number**	**IL-1β**
Control group	(n = 21)	1.19 ± 0.64
ACS group	(n = 21)	1.48 ± 0.57
*P* value	-	0.562

## Discussion

 In this study, the histological characteristics of soft tissue after horizontal ridge augmentation surgery showed that the number of fibroblasts in the ACS group was significantly higher than in the control group. ACS also significantly increased the amount of collagen in soft tissue compared to the control group. However, the number of inflammatory cells in the two groups was similar, and the expression of the IL-1 gene in the ACS and control groups were not significantly different. In the preparation of ACS, the incubation phase and the absence of anticoagulants and activators such as Ca21 or cells result in the main difference between ACS and other blood-derived products. Although they all contain platelets, only ACS absorbs the products of blood mononuclear cells that are synthesized during prolonged in vitro incubation.^9^ For example, Meijer et al^12^ showed that a large proportion of IL-1Ra in ACS is induced by de novo synthesis. Other important products are also likely to be synthesized during this period. Therefore, they might be different from other blood-derived products.

 Autologous blood tissue contains important growth factors. Platelets are essential blood components that play a significant role in these factors. Platelet activation stimulates degranulation and sequential release of trophic factors that affect wound healing and angiogenesis.^12^ Dutta et al^14^ introduced PRP as a biocompatible autologous product that significantly improved soft tissue healing and bone regeneration and increased bone density in extraction cavities. According to Kobayashi et al,^15^ PRP can induce more soft tissue regeneration by significantly increasing the stimulation of gingival fibroblast behavior compared to PDL cells or osteoblasts.

 Furthermore, some studies have shown the positive effects of laser therapy in soft tissue repair. In this regard, Fırat et al^16^ showed the positive effects of low-level laser therapy in epithelialization and wound healing after gingival surgery. Frozanfar et al^17^ also showed that LLLT stimulated the proliferation of human gingival fibroblasts and the expression of the type I collagen gene in vitro. In the present study, the biomolecular analysis indicated that the incidence of IL-1 was similar in both the control and intervention groups. Wehling et al^18^ used ACS in the mid-1990s as a rapid, practical, and relatively inexpensive tool to produce IL-1Ra for topical and therapeutic applications in musculoskeletal diseases. During this period, a 2-fold increase in IL-4, IL-10, and IL-13 occurs without an increase in proinflammatory cytokines IL-1b and TNF-α, according to a study by Kobayashi et al.^15^ Gingival fibroblasts cultured with PRP increased TGF-β, PDGF-B, and COL1 mRNA levels. Some studies have examined the effect of ACS on animals. In this regard, Velloso Alvarez et al^19^ showed that ACS is a strategy to improve the clinical symptoms of horses with osteoarthritis so that 50% ACS increases IL-10 expression and decreases IL-1β in cartilage. One of the limitations of the present study is the lack of examination of the initial thickness of soft tissue, and it is hoped that this parameter will also be measured in future studies.

## Conclusion

 In the present study, the amount of collagen and the number of fibroblasts increased in the ACS group compared to the control group, but the number of inflammatory cells in the ACS group did not show a significant difference from the control group. Also, gene expression (IL-1) did not show a significant difference in the ACS group compared to the control group.

## Acknowledgments

 We acknowledge the Periodontology Department, Faculty of Dentistry, for their assistance.

## Availability of data

 None.

## Competing Interests

 Adileh Shirmohammadi is the editor-in-chief of JAPID at the time of publication. The authors declare that they have no other competeing interests with regards to authorship and/or publication of this work.

## Ethical Approval

 This research was approved by the Research Ethics Committee of the Faculty of Dentistry under the code IR.TBZMED.REC.1400.353.

## Funding

 This work was supported by the Vice-Chancellor for Research, Faculty of Dentistry, Tabriz University of Medical Sciences, Tabriz, Iran.
